# Compatibility and Synergistic Effects of Plant Biostimulants and Commercial Entomopathogenic Fungi for *Diaphorina citri* Pathogenicity

**DOI:** 10.3390/insects17090947

**Published:** 2026-09-11

**Authors:** Éder M. Carrilho, Paulo S. G. Cremonez, Luciano M. de Oliveira, Fernando T. Hata, Maurício U. Ventura, Humberto G. Androcioli

**Affiliations:** 1 Agronomy Departament, Universidade Estadual de Londrina (UEL), Londrina 86057-970, PR, Brazil; eder.m.carrilho@uel.br (É.M.C.); mventura@uel.br (M.U.V.); 2 Department of Entomology and Plant Pathology, Auburn University, Auburn, AL 36849, USA; cremonez@auburn.edu; 3 Entomology Laboratory, Instituto de Desenvolvimento Rural do Paraná IAPAR-EMATER (IDR-Paraná), Londrina 86047-902, PR, Brazil; lucianomendes.oliveira@idr.pr.gov.br; 4 Agronomy Departament, Universidade Estadual de Maringá (UEM), Maringá 87020-900, PR, Brazil; fthata2@uem.br

**Keywords:** *Diaphorina citri*, citrus greening disease, entomopathogenic fungi, silicon dioxide, chitosan

## Abstract

The Asian citrus psyllid (ACP), *Diaphorina citri*, is the primary vector of Huanglongbing, a devastating disease threatening global citrus production. While chemical insecticides dominate control strategies, environmental and resistance concerns necessitate sustainable alternatives like Integrated Pest Management. This study evaluated the efficacy of commercial entomopathogenic fungi (EPF) formulations (*Beauveria bassiana*, *Metarhizium anisopliae*, and *Cordyceps javanica*) alone or combined with plant biostimulants (PBS; silicon dioxide and chitosan) across six greenhouse bioassays. Under hot, dry stress conditions (34 °C, 35% RH), stand-alone and PBS-paired EPF applications failed to induce significant *D. citri* mortality, highlighting environmental microclimate limitations. Conversely, under optimal controlled conditions (26 °C, 67% RH), biological treatments significantly increased mortality compared to untreated controls. Notably, the addition of PBS containing silicon dioxide and D-glucosamine into the spray did not exhibit antagonistic effects on fungal performance; instead, it significantly enhanced ACP mortality and survival reduction in several trials. While synthetic chemical control provided the fastest and highest initial mortality, these results demonstrate that tank-mixing commercial EPFs with silicon-dioxide-chitosan biostimulants is a compatible, viable, and highly promising strategy to optimize sustainable vector management under permissive environmental conditions.

## Simple Summary

The Asian citrus psyllid poses a major threat to global citrus production, driving interest in sustainable alternatives to synthetic chemical insecticides. To find safer options, the effectiveness of beneficial fungal treatments applied both on their own and combined with plant biostimulants was evaluated. This study shows that temperature and humidity play a pivotal role in treatment success: under hot, dry stress conditions, the fungal applications failed to significantly reduce pest populations. However, when applied in favorable environmental conditions, biological treatments significantly boost insect mortality. Notably, mixing the biostimulants into the fungal spray caused no harm to fungal performance and actually improved pest control in several tests. Although synthetic chemicals still provide the fastest initial pest reduction, combining beneficial fungi with plant biostimulants represents a viable and effective strategy for sustainable citrus crop protection under suitable weather conditions.

## 1. Introduction

The Asian citrus psyllid (ACP), *Diaphorina citri* Kuwayama, 1908 (Hemiptera: Liviidae), is considered the most significant pest in citrus production due to its role as the primary vector of Huanglongbing (HLB), also known as citrus greening disease [1]. The initial symptoms of HLB typically include leaf chlorosis on a single branch or within a localized section of the tree canopy. As the disease progresses, fruits exhibit reduced size, remain green upon maturation, display morphological deformities, and develop a bitter taste; in some cases, premature fruit drop may occur. In its chronic stage, HLB leads to severe defoliation and branch dieback [2]. Notably, adult psyllids can disperse over long distances, contributing to the rapid spread of the disease [3].

At present, chemical insecticides remain the primary method for controlling *D. citri* populations [4]. However, growing concerns regarding environmental imbalances and potential health hazards associated with these compounds have driven the search for safer and more sustainable alternatives [5]. Among the most promising approaches is Integrated Pest Management (IPM), which emphasizes ecological sustainability by combining multiple control strategies to reduce reliance on chemical inputs. Within IPM, Augmentative Biological Control (ABC) has gained increasing attention [6].

Several biological control agents are commercially available for use in ABC programs targeting various agricultural pests. Among these, entomopathogenic fungi (EPFs) represent a promising group of microbial control agents due to their unique mode of action, which involves direct contact with the host cuticle, penetration, and subsequent infection leading to insect mortality [7]. EPFs are effective across multiple developmental stages of the target insect and are considered safe for humans, animals, and plants [8]. In the case of ACP, several EPF species have demonstrated high pathogenicity, including *Isaria fumosorosea*, *Beauveria bassiana*, *Hirsutella citriformis*, *Lecanicillium lecanii*, *L. muscarium*, *L. longisporum*, *Metarhizium anisopliae*, and *M. brunneum* (Ascomycota: Hypocreales) [9].

Despite their advantages, the field performance of EPFs can be limited by environmental constraints, particularly exposure to ultraviolet radiation and elevated temperatures. These abiotic stressors can significantly impair fungal effectiveness by negatively impacting spore germination, survival, reproduction, dispersal, and virulence [10].

Plant biostimulants (PBS) have been proposed to increase crop yields and alleviate biotic and abiotic stress across several crops through various mechanisms. Currently, PBS usage has become prevalent, and a future increase in consumption is expected under ongoing climate change scenarios. Particularly, the combination of chitosan and silicon dioxide has been shown to improve plant photosynthesis and antioxidant defenses, making them more resilient to drought [11,12,13] and high temperatures [14,15]. Previous studies also suggested that the performance of EPFs could be enhanced, promoting insect pest mortality when combined with chitosan or silicon dioxide [16,17,18,19,20]. If confirmed, these improvements would be highly significant due to the urgent need to control disease vectors rapidly and efficiently.

Under typical orchard and cropping conditions, farmers often apply chemical mixtures within the same spray tank to avoid additional operational costs. However, these mixtures must be evaluated beforehand to assess potential antagonistic or additive effects. Experimentation on ACP under field conditions is challenging due to active disease transmission, which complicates the use of unprotected control treatments. Therefore, this study aimed to evaluate bioassay methods for assessing *D. citri* mortality using commercial EPF formulations, with a specific focus on evaluating mortality when these biological agents are combined with PBS in the spray solution.

## 2. Materials and Methods

**Figure 1:**
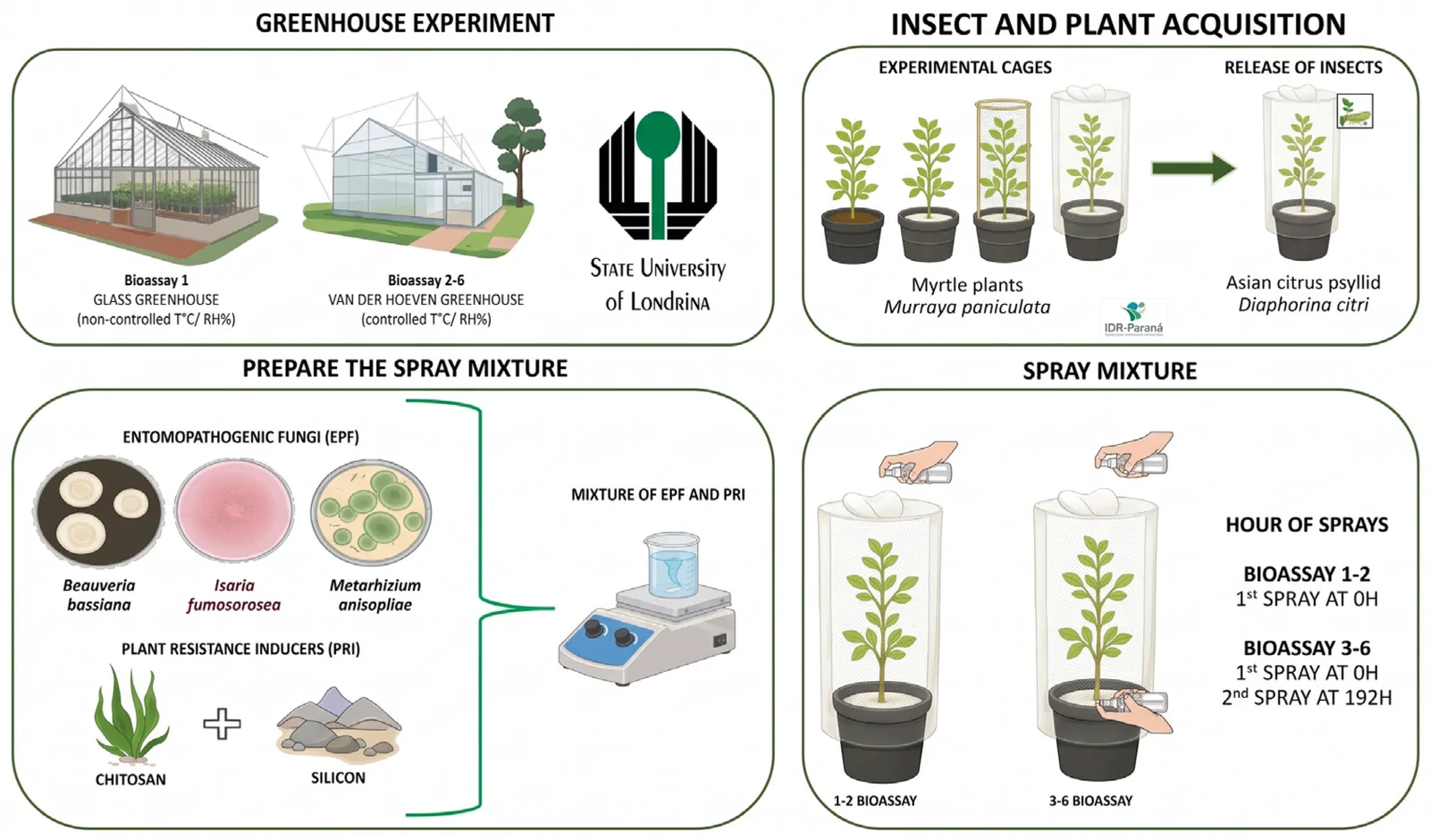
Schematic representation of the greenhouse bioassay workflow for *Diaphorina citri* management, illustrating the experimental setup, insect acquisition on *Murray paniculata*, spray mixture preparation using entomopathogenic fungi (*Beauveria bassiana*, *Isaria fumosorosea*, and *Metarhizium anisopliae*) combined with plant resistance inducers/biostimulants (chitosan and silicon dioxide), and application schedules.

Bioassays were conducted in greenhouses at the State University of Londrina—UEL (23°20′22.6″ S, 51°12′36″ W) located in the municipality of Londrina, Paraná State, Brazil (Figure 1). According to the Köppen–Geiger classification, the climate is categorized as Cfa, a hot-humid subtropical climate [21]. Temperature and relative humidity were monitored using a digital thermo-hygrometer (AKSO^®^ model AK28, Los Angeles, CA, USA) for both greenhouses, with the non-climate-controlled greenhouse (34 ± 2 °C, RH ≈ 35%) and the climate-controlled greenhouse, Van Der Hoeven model (26 ± 2 °C, RH ≈ 67%) (the temperature and relative humidity data are presented again below for each specific greenhouse). For insect rearing and bioassays, uninfected plants of orange jasmine (*Murraya paniculata*), an alternative myrtle host, were utilized [22].

Prior to setting up the trials, unsexed adult insects (1:1 expected sex ratio) were obtained from an established colony maintained from the Entomology Laboratory at the Instituto de Desenvolvimento Rural do Paraná–IDR-Paraná (23°21′21″ S, 51°09′51″ W). This colony is certified free of *Candidatus* Liberibacter asiaticus (CLas-free) and maintained under a standardized mass-rearing protocol, using orange jasmine (*Murraya paniculata*) as host plants under the controlled conditions of 26 ± 2 °C, 80 ± 10% RH, and natural day light cycles, that allows for precise age-cohort identification. Adult insects collected from 7 to 10 days post-emergence from rearing cages using a vacuum pump adapted from an entomological aspirator and transferred into 50 mL conical centrifuge tubes capped with a fine mesh secured by a rubber band to ensure proper ventilation. The insects were subsequently released into the bioassay cages and allowed to acclimate to a single host plant 24 h prior to spraying.

The experimental treatments included the following commercially formulated EPF insecticides: Boveril^®^ (*Beauveria bassiana* strain ESALQ PL63, BOV); Metarril^®^ (*Metarhizium anisopliae* strain ESALQ E9, MET); and Octane^®^ (*Cordyceps fumosorosea*, syn. *Isaria fumosorosea*, strain ESALQ 1296, OCT). These were applied either alone or associated with plant biostimulants (PBS): Tech Crop Power Zn^®^ (D-glucosamine from chitosan + Zn, CHI) and Tech Crop Si^®^ (biogenic calcium carbonate + silicon dioxide, SI), alongside the organosilicon adjuvant Silwet^®^ (AJV). A standard chemical control treatment using the synthetic insecticide Ampligo^®^ (Lambda-cyhalothrin + Chlorantraniliprole, CHE) and an untreated control (water only, UTC) were also included (Table 1).

The bioassays were conducted using cages constructed from malleable steel frames and voile fabric sleeves, featuring a sheet of white filter paper at the bottom to facilitate the counting of dead insects (Figure 1).

For the treatment solutions, the pH of water alone, water + CHI, water + SI, and water + CHI + SI was measured using a QUIMIS (model Q400A) benchtop pH meter. The pH of the spray solution containing PBS was 6.9. According to the EPF manufacturers, the suitable mixture pH ranges from 5.0 to 8.5. CHI and SI (PBS) were pre-mixed to form a single solution. This step is critical for managing ionic gelling resulting from the reaction between the sodium alginate polysaccharide (present in CHI) and soluble calcium [23]. Finally, the EPFs were added to the mixture, minimizing incompatibility caused by physicochemical alterations [24].

For Bioassays 1 and 2, a single spray application was performed in the late afternoon using a 500 mL transparent plastic hand-held trigger sprayer (Guarany^®^, model Leve Utrajet) (São Paulo, Brazil), fitted with an adjustable nozzle, set to a fine mist, delivering 5 mL of the treatment mixture per plant in a single top-to-bottom pass. For Bioassays 3–6, a standardized dual-application protocol was implemented following the manufacturers’ recommendations, with the first application performed 24 h after insect release and the second application conducted 192 h post-release (24 h after the 168 h evaluation). In each application of Bioassays 3–6, a total volume of 10 mL per cage (containing one host plant) was delivered using two directional passes: 5 mL from top to bottom and 5 mL from bottom to top (Figure 1). This bidirectional spraying was designed to optimize conidial deposition and attachment to both the dorsal anterior and abdominal regions of *D. citri*, thereby increasing infection probability [25]. Prior to all bioassays, the delivered spray volume was calibrated by determining the mean volume released per trigger actuation using a graduated cylinder. Across all experiments, ACP adults were sprayed directly while resting on the host plants following a 24 h acclimation period, ensuring direct contact with the spray droplets.

To evaluate treatment efficacy under distinct environmental scenarios, two initial independent bioassays were conducted. Bioassay 1 was performed in an un-climatized glass greenhouse under stressful abiotic conditions (34 ± 2 °C, RH ≈ 35%) to assess the overall performance of four treatments (UTC, CHE, BOV, and BOV + PBS; three replicates, n = 10) under elevated temperature and low relative humidity. Bioassay 2 was conducted independently in a climate-controlled greenhouse (Van Der Hoeven model) under optimal conditions (26 ± 2 °C, RH ≈ 67%) following the same treatment protocol, serving to establish baseline efficacy without abiotic stress; all subsequent bioassays (3–6) were maintained under these optimal conditions (Figure 1).

All the descriptions from bioassay 3 to 6 are in Table 2. Bioassay 3 consisted of eight treatments (UTC, CHE, BOV, BOV + PBS, MET, MET + PBS, OCT, and OCT + PBS) applied in two sequential sprays, evaluated to 25 adult Asian citrus psyllids per cage (75 insects per treatment). While Bioassay 4 evaluated the addition of the silicone dioxide adjuvant Silwet^®^ at a concentration of 0.05% *v*/*v* (AJV) across all biological treatments, comprising eight treatments in total: UTC, CHE, BOV + AJV, BOV + PBS + AJV, MET + AJV, MET + PBS + AJV, OCT + AJV, and OCT + PBS + AJV. This bioassay was conducted with 30 adult ACP per cage (90 insects per treatment). Bioassay 5 re-evaluated combinations of biological agents without adjuvant using eight treatments (UTC, CHE, BOV, BOV + PBS, MET, MET + PBS, OCT, and OCT + PBS), with 30 adult *D. citri* per cage (90 insects per treatment). Bioassay 6 assessed six treatments (UTC, PBS, BOV, BOV + PBS, OCT, and OCT + PBS) to evaluate key fungal and microsphere combinations, with 50 adult ACP per cage (150 insects per treatment).

The number of adult *Diaphorina citri* per cage varied across bioassays (25, 30, and 50 adults) due to progressive optimization of experimental power and insect availability. In initial trials (Bioassay 3), 25 insects per cage were used based on standard rearing yield. In subsequent trials (Bioassays 4–5), the density was increased to 30 adults per cage to improve statistical precision while maintaining optimal cage capacity. For Bioassay 6, the density was further increased to 50 adults per cage to increase statistical power for detecting fine differences in survival curves, while ensuring that overcrowding stress did not artificially inflate control mortality (UTC remained within acceptable threshold limits).

Mortality assessment was achieved by collecting dead insects from the white filter paper using fine-tipped entomological forceps. Dead insects were identified by their curved integuments, legs and mouthparts detached from the host plants, and an overall reduced body volume due to dehydration without visible signs of external sporulation [26]. To establish precise daily survival curves, insect mortality was monitored daily (every 24 h) post-spraying up to 240 h for Bioassays 1 and 2, and up to 480 h for Bioassays 3–6.

Survival times among different treatments were estimated using the Kaplan–Meier method based on daily observation intervals, and differences between the resulting survival curves were evaluated using the global Log-Rank test. The Log-Rank test identified significant differences between treatments by calculating chi-square values and corresponding *p*-values. Additionally, pairwise comparisons between treatments were performed using the Log-Rank test adjusted for multiple comparisons via the Benjamini–Hochberg method. Survival times were measured in hours, and mean survival times were calculated for each treatment group. All survival calculations were performed using the survival package in R software (version 4.5.2), deploying the *survfit*() function for Kaplan–Meier curves, *survdiff*() for the global Log-Rank test, and *pairwise_survdiff*() for adjusted pairwise comparisons. Results were considered statistically significant at p<0.05.

To evaluate cumulative mortality at specific operational timepoints (accounting for fungal incubation), mean mortality data were specifically analyzed at 24 and 240 h (Bioassays 1 and 2) and at 24, 192 (24 h after the second spraying), and 480 h (Bioassays 3–6). These data were subjected to Shapiro–Wilk’s normality test and Bartlett’s test for homogeneity of variance. Data meeting ANOVA assumptions were compared using Tukey’s honest significant difference (HSD) test. When assumptions were violated, data were analyzed using the non-parametric Kruskal-Walli’s test. These analyses were conducted utilizing the *DIC*() function from the AgroR package [27]. This function is designed for analyzing qualitative, uniform agricultural experiments in a completely randomized design with multiple assessments over time, without treating time as an interactive factor. Results were considered statistically significant at *p* < 0.05.

## 3. Results

**Figure 2:**
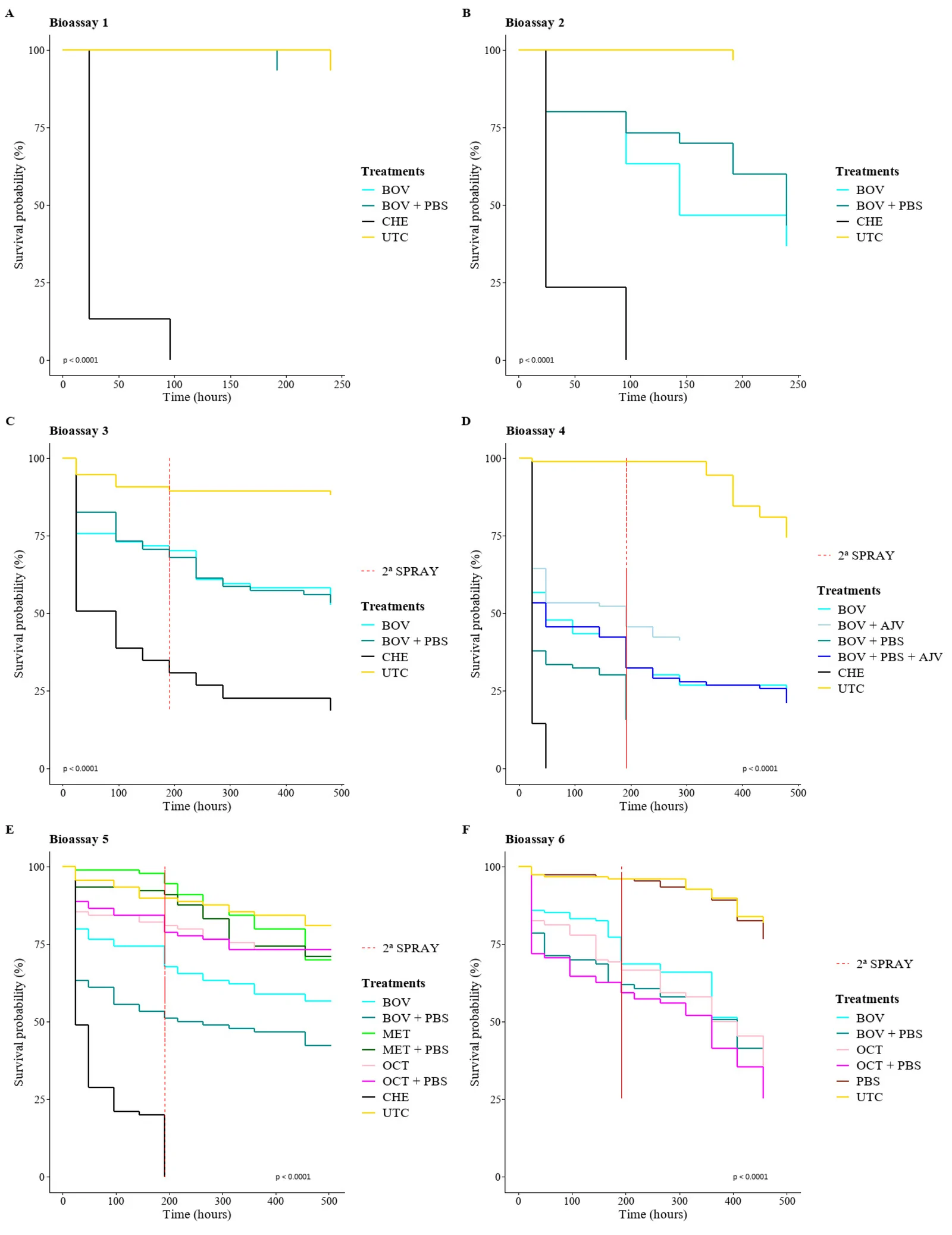
Kaplan–Meier survival curves of *Diaphorina citri* adults exposed to entomopathogenic fungi (EPF)-based treatments with or without plant biostimulants [silicon dioxide and chitosan (PBS)] across six greenhouse bioassays, represented as bioassay 1 (**A**), bioassay 2 (**B**), bioassay 3 (**C**), bioassay 4 (**D**), bioassay 5 (**E**) and bioassay 6 (**F**). The red dashed line indicates the second spray application in bioassays 3–6. All comparisons were statistically significant (Log-Rank test, *p* < 0.05).

Across all completed assays, pairwise comparisons revealed significant differences among treatments based on Kaplan–Meier survival curves (chi^2^ = 136, *p* < 0.05; chi^2^ = 75.6, *p* < 0.05; chi^2^ = 83.8, *p* < 0.001; chi^2^ = 226, *p* < 0.0001; chi^2^ = 351, *p* < 0.001; chi^2^ = 184, *p* < 0.0001 for bioassays 1 through 6, respectively) (Figure 2).

**Figure 3:**
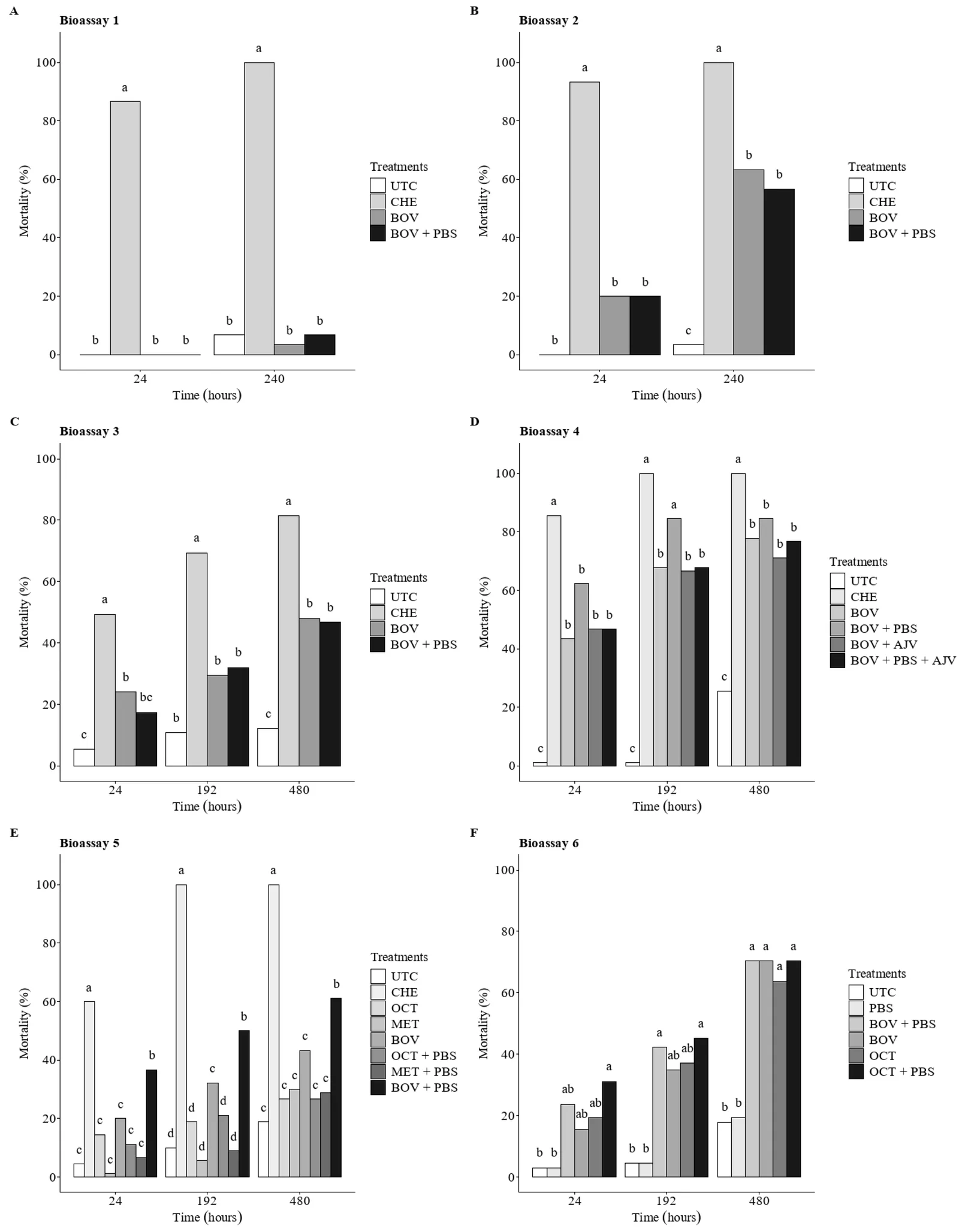
Mortality (%) of *Diaphorina citri* adults at different time points in Bioassays 1–2 (24 and 240 h) and Bioassays 3–6 (24, 192, and 480 h), following exposure to various EPF-based treatments with or without plant biostimulants [silicon dioxide and chitosan (PBS)] across six greenhouse trials, represented as bioassay 1 (**A**), bioassay 2 (**B**), bioassay 3 (**C**), bioassay 4 (**D**), bioassay 5 (**E**) and bioassay 6 (**F**). Bars with different letters indicate statistically significant differences according to Tukey’s test (*p* < 0.05).

In the second bioassay, mirroring the first, significantly higher mortality was observed for CHE compared to all other treatments, with observed events (30) exceeding expected counts (11). The BOV and BOV + PBS treatments also achieved higher mortality rates than the untreated control (*p* < 0.05), in which observed mortality events fell below expectations. Ultimately, the CHE treatment led to the highest overall mortality, while both BOV and BOV + PBS significantly outperformed the UTC group (Figure 3B).

Mortality was significantly higher for the CHE treatment than all remaining groups (*p* < 0.05) in the third bioassay (Figure 3C). Following the initial application (24 has), the highest mortality was observed in CHE, followed by intermediate rates in BOV and BOV + PBS, and the lowest in UTC. After the second application (indicated by the red line in Figure 2C) at 192 and 480 has, the same relative hierarchy among treatments persisted, though with overall increased cumulative mortality.

For the fourth bioassay, trends closely matched prior experiments. The CHE treatment again caused the highest mortality, and the fungal treatments remained superior to the UTC. In the second assessment window, the BOV + PBS combination yielded significantly higher mortality than BOV alone and the remaining treatments (Figure 2D and Figure 3D). Formulations incorporating the silicon dioxide adjuvant behaved similarly to matching treatments without the adjuvant. In the fifth experiment, besides the CHE treatment, only the BOV and BOV + PBS groups significantly surpassed UTC mortality levels across assessments (Figure 2E). Notably, higher mortality was observed for BOV + PBS than for BOV alone (Figure 3E).

During the sixth experiment, assessments were completed at 24, 192, and 480 h. Treatments featuring standalone biological agents displayed survival rates like the UTC and PBS-only groups (Figure 2F). However, the OCT + PBS combination (at 24, 192, and 480 h) and the BOV + PBS combination (at 192 and 480 h) significantly diminished insect survival compared to both UTC and PBS alone. By the final assessment, biological treatments paired with biostimulants successfully outpaced both the untreated control and the standalone PBS treatment (Figure 3F).

## 4. Discussion

The use of caged myrtle plants provided a standardized setup to compare the applied treatments within each assay. Under the conditions of bioassay 1, treatments utilizing the commercial formulation of *Beauveria bassiana* (BOV and BOV + PBS) did not result in a significant increase in *D. citri* mortality compared to the control (Figure 2A and Figure 3A). The elevated temperature and low relative humidity recorded during this period (34 °C ± 2 °C, RH ~ 35%) align with thresholds known to severely impair entomopathogenic fungal (EPF) development, where temperatures exceeding 31.5 °C combined with low RH typically hamper EPF efficacy [28,29]. Conversely, in bioassay 2, conducted under lower thermal and higher moisture conditions (26 °C ± 2 °C, RH ~ 67%), BOV and BOV + PBS treatments yielded significantly higher mortality than the UTC (Figure 2B and Figure 3B), a trend consistently observed across bioassays 3–6 (Figure 2C–F and Figure 3C–F).

However, because bioassays 1 and 2 were conducted in different greenhouse facilities and at distinct experimental times, environmental factors (temperature and RH) were confounded with temporal and spatial variations. Furthermore, as a PBS-only control was not included in bioassay 1 and no formal interaction analysis (fungus × adjuvant × environment) was performed, the present data cannot definitively establish the protective limits of PBS under extreme stress, nor distinguish physical or biological compatibility from additive effects on mortality. To rigorously test the protective capacity of PBS on EPFs, including potential mechanisms such as enhanced conidial thermal tolerance, cuticle penetration, or germination, future studies utilizing controlled climate chambers and complete factorial designs are required. Nevertheless, the lack of efficacy observed under the high-stress conditions of bioassay 1 highlights the practical importance of aligning EPF field deployments with favorable microclimatic windows, particularly given the increasing frequency of heatwaves in citrus-producing regions [30].

Overall, mortality rates in the EPF treatments were lower than those in the synthetic CHE treatment (Figure 2A and Figure 3A to Figure 2E and Figure 3E). EPFs also required a longer latency period to reduce survival, as little to no mortality increases were observed within the first 24 h. These timeline lags represent clear limitations when using standalone EPFs for ACP suppression. For vector insects, rapid mortality is ideal to disrupt plant disease transmission. Consequently, integrating compatible EPFs with synthetic chemical insecticides has been proposed to manage sap-sucking vectors. Combining compatible chemical and biological components has been shown to extend the window of biological activity against target pests [31].

Furthermore, even when immediate mortality is not achieved, vector transmission dynamics may still be altered. For instance, commercial formulations of *C. fumosorosea* disrupt the feeding behavior of Asian citrus psillid, specifically impairing phloem-probing activities, as demonstrated via electrical penetration graphs (EPG) at 30 and 96 h post-spraying [32].

The inclusion of an organosilicon adjuvant in bioassay 4 did not enhance insect mortality (Figure 2D and Figure 3D). However, supplementing the biological formulation with PBS (BOV + PBS) significantly improved efficacy compared to BOV alone at 192 h. This lack of enhanced performance from the silicon dioxide surfactant contrasts with prior studies that reported increased mortality in the two-spotted spider mite, *Tetranychus urticae*, when surfactants were mixed with fungal spores [16]. Reductions in survival due to the addition of PBS to the *B. bassiana* formulation were also evident in bioassay 5 across all three assessment points (Figure 2E and Figure 3E). Finally, in bioassay 6, adding PBS to the biological treatments provided a marginal efficiency boost, rendering the combination significantly superior to the untreated control, whereas the EPF-only applications fell into an intermediate, non-significant tier (Figure 2F and Figure 3F).

The commercial PBS evaluated in this study contains silicon dioxide and chitosan. Synergies between silicate fertilizers and entomopathogenic fungi have been documented to help break down lipid barriers in the insect cuticle, facilitating fungal penetration and colonization [19], while simultaneously improving spore thermal resistance, virulence, and pathogenicity [18]. Beyond silicon dioxide, the D-glucosamine units within chitosan are known to improve the environmental availability and stability of chemical and botanical insecticides [5,33].

Direct insecticidal properties of chitosan solutions have also been recorded against *Hyalopterus pruni* (Hemiptera: Aphididae) [34]. When exposed to chitosan-enriched media, EPFs, particularly *B. bassiana*, exhibit enhanced sporulation while maintaining high viability and pathogenicity. This occurs because the fungi can produce chitin-degrading enzymes and utilize chitosan derivatives and byproducts as supplemental nutrients [17].

To build on these findings, future research should systematically evaluate the compatibility and efficacy of entomopathogenic fungi (EPF) and plant biostimulant (PBS) mixtures across a broader matrix of precise temperatures and relative humidity levels within controlled climatic chambers. This would explicitly define the microclimatic thresholds required to safeguard fungal spores from environmental degradation, which is increasingly critical under current climate change scenarios and frequent heatwaves. Finally, large-scale open-field trials are necessary to assess the real-world operational viability, residual persistence, and multi-season economic benefits of tank-mixing these biological agents for integrated citrus pest management.

## 5. Conclusions

The caged-plant bioassay methodology serves as an effective framework for evaluating insecticide options against *D. citri*. While synthetic chemical insecticides provide faster and more robust initial mortality, integrating commercial plant biostimulants containing silicon dioxide and D-glucosamine into entomopathogenic fungal spray mixtures does not hinder biological performance. Instead, this combination enhanced Asian citrus psillid mortality in several experiments, demonstrating that tank-mixing these components is a viable and potentially desirable strategy for integrated vector management.

## Figures and Tables

**Table 1:** Commercial products, active ingredients or viable conidia concentrations, formulations, and doses evaluated in greenhouse bioassays against *Diaphorina citri* on caged plants. Abbreviations: SC: Suspension concentrate; WP: Wettable powder; SL: Soluble concentrate.

Code	Commercial Name^TM^ (Manufacture)	Description	Active Ingredients or Viable Conidia Concentration	Formulation	Dose Bioassay 1 (mL or g L^−1^)	Dose Bioassays 2–6 (mL or g L^−1^)
UTC	-	Untreated check	-	-	-	-
CHE	Ampligo (Syngenta)	Lambda-cyhalothrin + Chlorantraniliprole	100 + 50	SC	0.1	0.1
BOV	Boveril (Koppert)	*Beauveria bassiana* (strain ESALQ PL63)	2.00 × 10^9^	WP	4.0	10.0
MET	Metarril (Koppert)	*Metarhizium anisopliae* (strain ESALQ E9)	1.39 × 10^8^	WP	3.0	3.0
OCT	Octane (Koppert)	*Isaria fumosorosea* (strain ESALQ 1296)	2.50 × 10^9^	SC	3.2	4.0
CHI	Tech Crop Power Zn (TechCrop)	D-Glucosamine from chitosan + Zn	53% Zinc Sulfate 43.5% Water 3.5% Amines	SC	1.5	1.5
SIL	Tech Crop Si (TechCrop)	Biogenic Ca (10.2%) + Silicon Dioxide	68.2% Diatomaceous Earth 31.8% Calcium Carbonate	WP	7.5	7.5
AJV	Silwet (UPL)	Non-ionic organosilicone surfactants	Polyalkyleneoxide modified heptamethyltrisiloxane (1000 g/L)	SL	-	0.5 (Bioassay 4)

**Table 2:** Summary of experimental design and operational parameters for Bioassays 3–6 evaluating the efficacy of biological agents against *Diaphorina citri* adults. Abbreviations: UTC = Untreated Control; CHE = Chemical Control; BOV = *Beauveria bassiana*; MET = *Metarhizium anisopliae*; OCT = *Cordyceps fumosorosea*; PBS = Plant biostimulant (silicon dioxide + chitosan); AJV = Silwet^®^ organosilicone adjuvant (0.05% *v*/*v*). Product concentrations for each biological agent and biostimulant are detailed in Table 1.

Bioassays	Treatment List	Product Concentration	Spray Volume	Application Timing	Replicate	Insects per Cage (Total)
3	UTC, CHE, BOV, BOV + PBS, MET, MET + PBS, OCT, OCT + PBS	See Table 1 for biologicals	10 mL (5 mL top-down + 5 mL bottom-up)	2 sprays (24 h and 192 h post-release)	3	25 (75)
4	UTC, CHE, BOV + AJV, BOV + PBS + AJV, MET + AJV, MET + PBS + AJV, OCT + AJV, OCT + PBS + AJV	Silwet^®^ at 0.05% *v*/*v* (AJV); See Table 1 for biologicals	10 mL (5 mL top-down + 5 mL bottom-up)	2 sprays (24 h and 192 h post-release)	3	30 (90)
5	UTC, CHE, BOV, BOV + PBS, MET, MET + PBS, OCT, OCT + PBS	See Table 1 for biologicals	10 mL (5 mL top-down + 5 mL bottom-up)	2 sprays (24 h and 192 h post-release)	3	30 (90)
6	UTC, PBS, BOV, BOV + PBS, OCT, OCT + PBS	See Table 1 for biologicals	10 mL (5 mL top-down + 5 mL bottom-up)	2 sprays (24 h and 192 h post-release)	3	50 (150)

## Data Availability

The raw data supporting the conclusions of this article will be made available by the authors on request.
